# Alpha particle therapy in glioblastoma: emerging biomarkers, mechanisms of response, and translational opportunities

**DOI:** 10.3389/fonc.2026.1940181

**Published:** 2026-09-04

**Authors:** Tugce Kutuk, Ece Atak, Marshall Harrell, Raju Raval, Sasha Beyer, Simeng Zhu, John Grecula, Fatemeh Fekrmandi, Sean S. Mahase, Khaled Dibs, Daniel Trifiletti, Raj Singh, James Bradley Elder, Kyle Wu, Pierre Giglio, Dukagjin M. Blakaj, Arnab Chakravarti, Joshua Palmer

**Affiliations:** 1 Department of Radiation Oncology, The Ohio State University Wexner Medical Center, Columbus, OH, United States; 2 Department of Radiation Oncology, Basaksehir Cam and Sakura City Hospital, Istanbul, Türkiye; 3 Department of Radiation Oncology, Mayo Clinic Florida, Jacksonville, FL, United States; 4 Department of Radiation Oncology, Lynn Cancer Institute, Baptist Health South Florida, Boca Raton, FL, United States; 5 Department of Neurological Surgery, The Ohio State University Wexner Medical Center, Columbus, OH, United States; 6 Division of Neuro-Oncology, Department of Neurology, The Ohio State University Wexner Medical Center, Columbus, OH, United States

**Keywords:** alpha dart, alpha-particle therapy, glioblastoma, radium-224, substance P, targeted alpha therapy (TAT)

## Abstract

Glioblastoma remains the most aggressive primary malignant brain tumor in adults. Disease progression is driven by multiple biological mechanisms, including glioma stem cell persistence, intratumoral heterogeneity, hypoxia-associated treatment resistance, enhanced DNA damage repair, immune evasion, and dynamic interactions within the tumor microenvironment. These factors contribute to therapeutic failure and highlight the need for novel treatment strategies and biomarker-driven approaches guiding patient selection and response assessment. Alpha particle therapy is a promising modality due to it’s high linear energy transfer (LET), short tissue range, and potent relative biological effectiveness (RBE), inducing complex DNA damage that is difficult to repair while limiting radiation exposure to surrounding normal tissue. Among emerging platforms, Diffusing Alpha-emitters Radiation Therapy (Alpha DaRT) enables sustained intratumoral delivery of alpha-emitting radionuclides, while targeted alpha therapies employ tumor-specific radiopharmaceuticals to selectively deliver highly cytotoxic radiation to malignant cells. Beyond direct tumor cell killing, accumulating evidence suggests alpha particle therapy targets several biological pathways implicated in glioblastoma progression, including DNA damage response signaling, glioma stem cell survival, hypoxia-driven adaptation, and immune suppression. These mechanisms generated growing interest in the identification of predictive and pharmacodynamic biomarkers capable of guiding treatment selection and monitoring therapeutic response. Candidate biomarkers include DNA repair-associated proteins, stemness-related markers, molecular alterations such as MGMT and IDH status, liquid biopsy analytes, and advanced imaging biomarkers. In this review, we summarize current alpha-emitting therapeutic strategies for glioblastoma, with particular emphasis on Alpha DaRT, and discuss emerging biological mechanisms of response, biomarkers associated with tumor progression and therapeutic sensitivity, and opportunities for biomarker-guided clinical implementation.

## 1 Introduction

Glioblastoma remains the most common and aggressive primary malignant brain tumor in adults (1). Despite advances in neurosurgery, radiation oncology, molecular diagnostics, and systemic therapy, outcomes remain poor. The current standard treatment entails maximally safe resection followed by concurrent radiotherapy with temozolomide, adjuvant temozolomide, and tumor treating fields for eligible patients; however, the 5-year overall survival (OS) rate remains only 9.8% (2–5). Approximately 90% of patients recur within 6 months of treatment completion, with most recurrences located within 2 cm of the original tumor site (6).

The limited efficacy of current therapies reflects the complex biology of glioblastoma. Glioma stem cells (GSCs) represent a particularly important therapeutic challenge because of their capacity for self-renewal, tumor repopulation, and resistance to both chemotherapy and radiotherapy (7, 8). These cells exhibit enhanced DNA damage repair mechanisms and can survive cytotoxic treatment, contributing to inevitable disease recurrence. In parallel, substantial intratumoral heterogeneity at the genomic, epigenetic, cellular, and spatial levels promotes the emergence of resistant subclones and limits the effectiveness of targeted therapies (9). The tumor microenvironment further contributes to treatment failure through immunosuppression, abnormal vascular architecture, metabolic reprogramming, and interactions between malignant cells and surrounding stromal and immune populations (10).

Despite advances in maximally safe resection, radiotherapy, temozolomide, and tumor treating fields for newly diagnosed disease, therapeutic options for recurrent glioblastoma remain limited. Current treatment recommendations emphasize individualized management with repeat surgical resection when feasible, systemic therapies such as bevacizumab, nitrosoureas, or regorafenib, selected cases of reirradiation, and enrollment in clinical trials whenever possible, as no salvage therapy has consistently demonstrated durable disease control or substantial improvements in OS (11–13). Consequently, there remains a significant unmet need for novel local therapies capable of overcoming radio resistance while minimizing additional radiation exposure to previously irradiated normal brain tissue.

**Figure 1:**
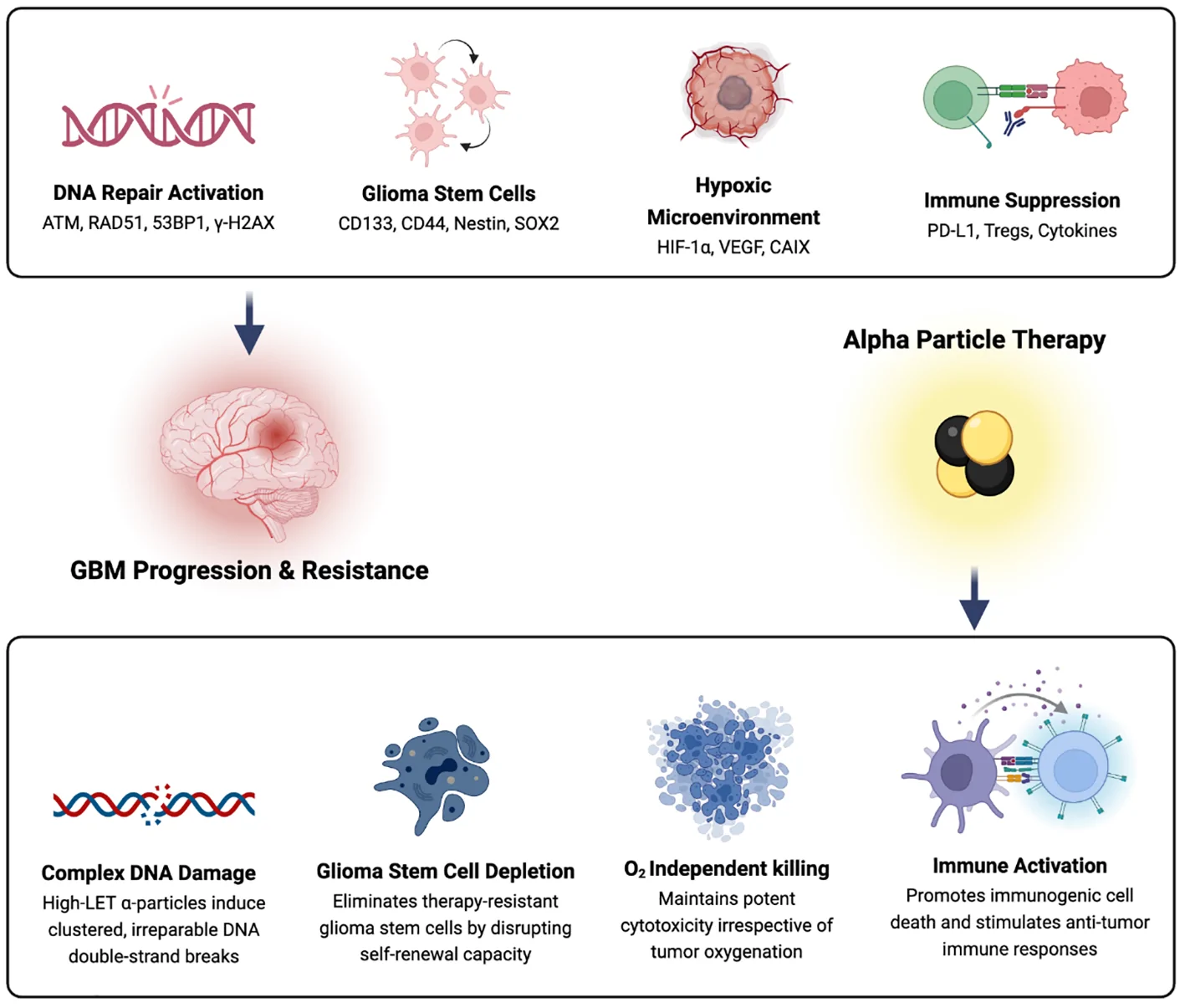
Biological mechanisms contributing to glioblastoma progression and therapeutic resistance, and the proposed mechanisms by which alpha-particle therapy may overcome these barriers. Glioblastoma progression is driven by enhanced DNA damage repair, glioma stem cell (GSC) maintenance, adaptation to hypoxic microenvironments, and immune suppression. High-linear energy transfer (LET) alpha-particle therapy induces complex, irreparable DNA double-strand breaks and may overcome these barriers through depletion of therapy-resistant GSC populations, maintenance of cytotoxic efficacy under hypoxic conditions, and promotion of immunogenic cell death with activation of innate and adaptive immune responses. With the exception of complex DNA damage induction, these mechanisms are supported primarily by preclinical studies and remain to be validated in clinical studies of glioblastoma. The pathways illustrated are adapted from the current understanding of glioblastoma biology and alpha-particle radiobiology (7, 8, 17–20). Created in BioRender. Atak, E. (2026) https://BioRender.com/af5v7ev.

Alpha particle therapy has emerged as a promising investigational strategy for glioblastoma because of its unique physical and biological characteristics. Alpha particles are helium nuclei that deposit large amounts of energy over short distances in tissue. Their high LET, typically ranging from 50 to 230 keV/μm, results in dense ionization tracks that induce complex and clustered DNA damage (14). Unlike the DNA lesions produced by conventional low LET radiation, these clustered double strand breaks are often irreparable, promoting mitotic catastrophe, apoptosis, and other forms of cell death. A single alpha particle traversal through the nucleus may be sufficient to permanently inactivate a tumor cell. The RBE of alpha radiation is three to seven times greater than that of photon or beta-particle irradiation and is substantially less dependent on oxygenation status and cell cycle phase, providing a strong radiobiological rationale for overcoming hypoxia-associated radio resistance and targeting therapy-resistant cellular populations. However, whether these biological advantages translate into improved clinical outcomes in patients with glioblastoma remains to be established (15, 16) (**Figure 1**).

Interest in alpha particle therapy for glioblastoma has increased with the development of novel delivery platforms capable of achieving highly localized radiation deposition. The convergence of advances in alpha particle delivery and an improved understanding of glioblastoma biology has created new opportunities to develop therapies that address key mechanisms of treatment resistance while enabling biomarker-driven patient selection. Current investigational approaches include Diffusing Alpha-emitters Radiation Therapy (Alpha DaRT), which uses interstitial ^224^Ra-containing sources, and targeted alpha therapy, which delivers alpha-emitting radionuclides to tumor-specific molecular targets, including neurokinin type-1 receptor-directed agents such as ^213^Bi-DOTA-Substance P and ^225^Ac-DOTA-Substance P (21–23). Although encouraging preclinical studies and early clinical experience with selected alpha-particle platforms support continued investigation, human clinical evidence in glioblastoma remains limited. In particular, the evidence supporting Alpha DaRT in GBM is currently confined to preclinical studies and an ongoing early-phase clinical trial, and its clinical efficacy has not yet been established. Beyond their direct cytotoxic effects, alpha particle therapies may influence several biological processes implicated in tumor progression and treatment resistance, including DNA damage response pathways, GSC maintenance, hypoxia-associated adaptation, immune evasion, and tumor microenvironment remodeling (24). These pathways are increasingly recognized not only as therapeutic vulnerabilities but also as potential sources of predictive and pharmacodynamic biomarkers. While established molecular markers such as MGMT promoter methylation and IDH mutation status provide important prognostic and therapeutic information in glioblastoma, additional biomarkers capable of predicting response to alpha particle therapy remain largely unexplored. Emerging candidates include DNA damage response proteins, stemness-associated biomarkers, homologous recombination repair signatures, circulating tumor DNA, extracellular vesicles, and advanced imaging biomarkers. In this review, we summarize the current biological rationale, preclinical evidence, and emerging clinical experience supporting alpha-particle therapy for glioblastoma, with particular emphasis on Alpha DaRT. We also discuss mechanisms of response and resistance, candidate biomarkers of therapeutic sensitivity, and the translational opportunities and challenges that must be addressed before these approaches can be incorporated into routine clinical practice.

## 2 Review methodology

This narrative review summarizes the current evidence on alpha-particle therapy for glioblastoma, with a particular focus on Alpha DaRT and biomarker-guided therapeutic strategies. A literature search was conducted using PubMed/MEDLINE, Embase, and ClinicalTrials.gov for articles published through July 2026. Search terms included combinations of “glioblastoma,” “alpha-particle therapy,” “targeted alpha therapy,” “Alpha DaRT,” “Diffusing Alpha-emitters Radiation Therapy,” “^213^Bi,” “^225^Ac,” “^211^At,” “Substance P,” “radiopharmaceutical therapy,” “biomarkers,” and “high linear energy transfer.” Relevant clinical studies, preclinical investigations, review articles, ongoing clinical trials, and selected conference abstracts describing emerging technologies or early clinical experience were included to provide a comprehensive overview of the field.

## 3 Alpha emitting therapeutic platforms for glioblastoma

**Figure 2:**
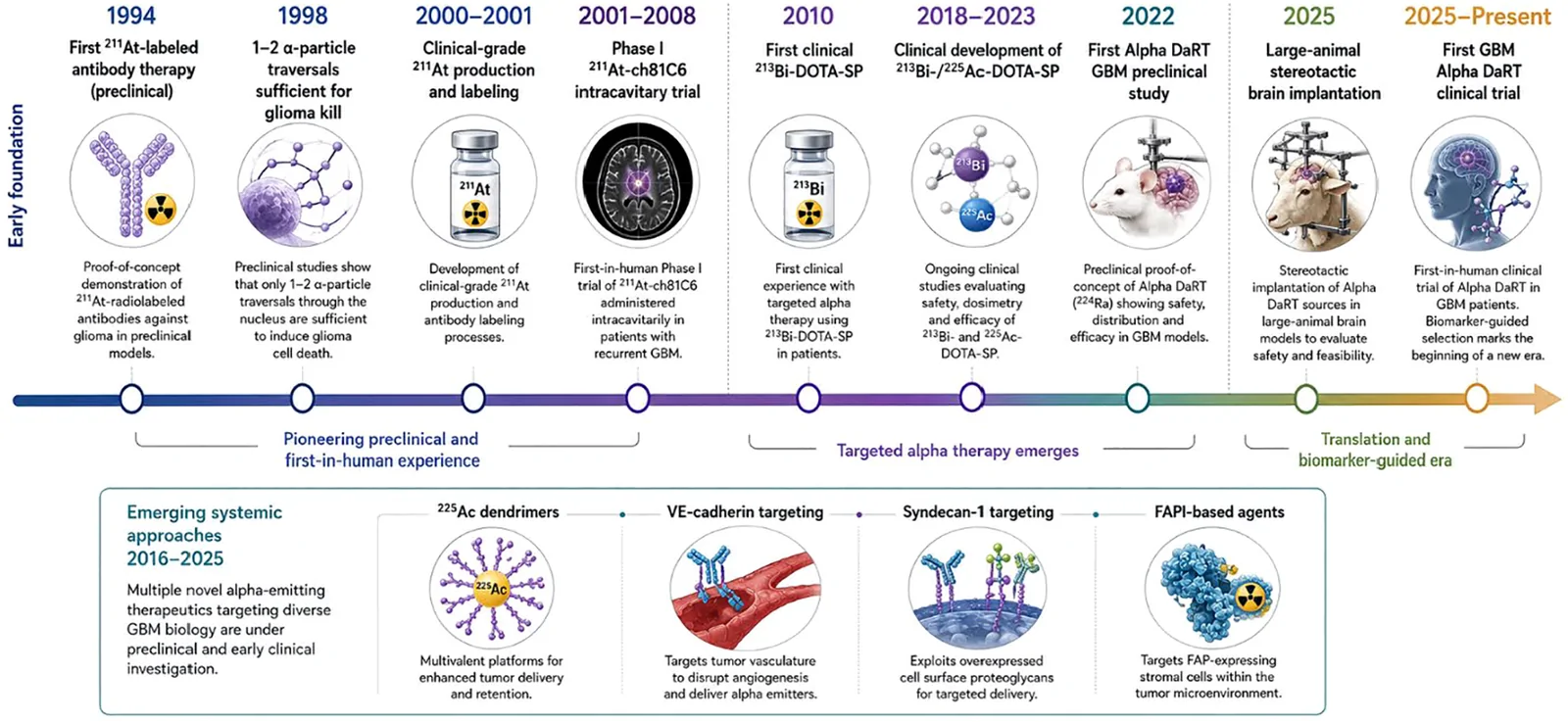
Chronological evolution of alpha-particle therapy for glioblastoma. GBM, glioblastoma; Alpha DaRT, Diffusing Alpha-emitters Radiation Therapy; FAPI, fibroblast activation protein inhibitor; Ac, actinium; At, astatine; Bi, bismuth; DOTA, tetraazacyclododecane tetraacetic acid.

**Figure 3:**
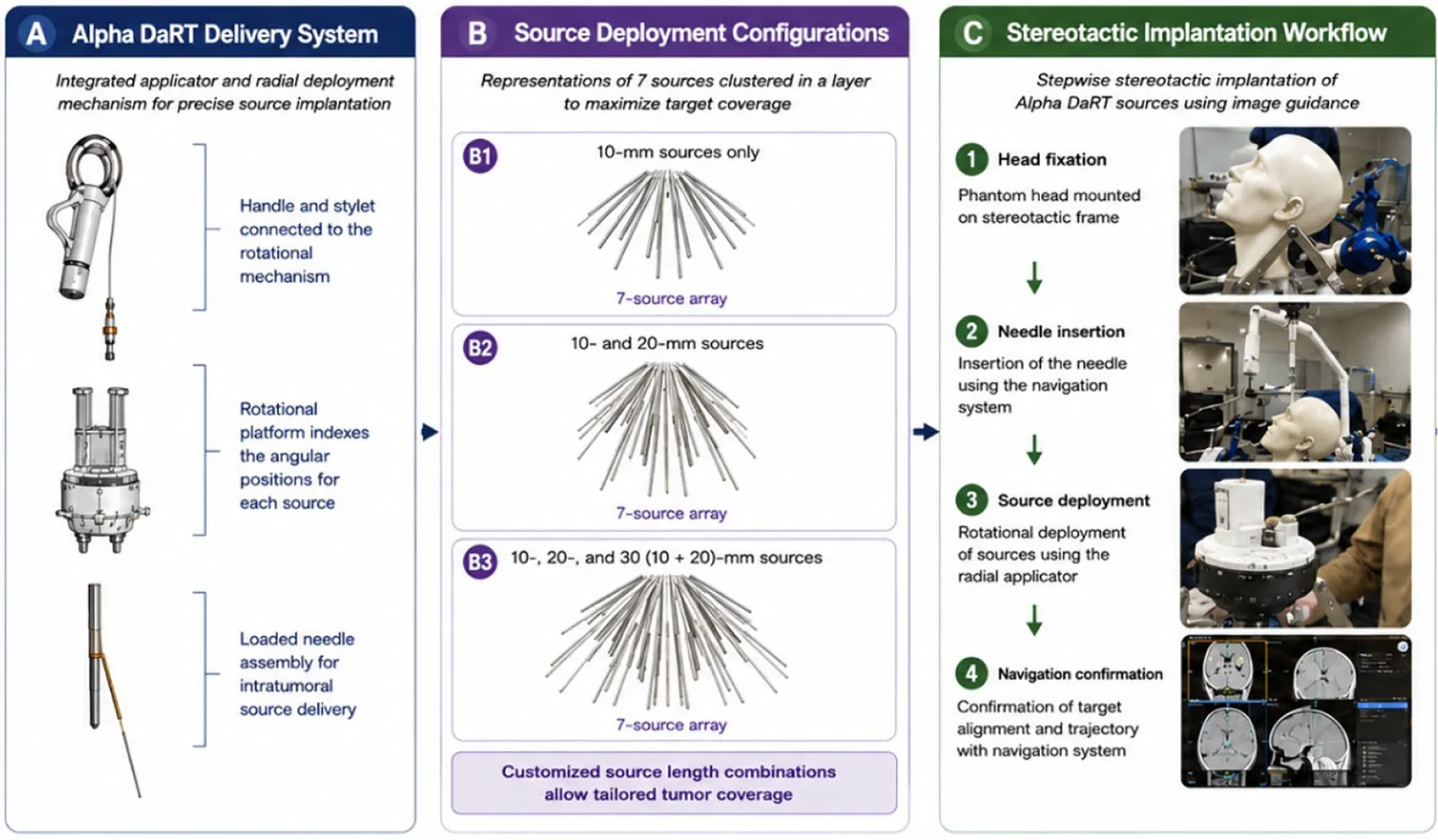
Alpha DaRT device, source configurations, and stereotactic intracranial implantation workflow. **(A)** Components of the Alpha DaRT delivery system, including the radial deployment applicator, rotational indexing platform, and loaded needle assembly used for intratumoral source implantation. **(B)** Representative source deployment configurations illustrating different combinations of 10-, 20-, and 30-mm ^224^Ra sources that can be customized to optimize target coverage based on tumor geometry. **(C)** Stepwise stereotactic implantation workflow demonstrating head fixation, image-guided needle insertion, radial source deployment, and navigation-based confirmation of source placement.

The evolution of alpha particle therapy for glioblastoma has progressed from early preclinical proof-of-concept studies using ²¹¹At-labeled antibodies to targeted alpha therapies, interstitial Alpha DaRT, and emerging systemic approaches. The major milestones in this development are summarized in **Figure 2**. Current alpha-particle therapeutic platforms for glioblastoma can be broadly categorized according to their delivery strategy into three groups (1): interstitial alpha therapy, exemplified by Alpha DaRT (2); locoregional targeted alpha therapy, in which radiolabeled agents are administered directly into the tumor or resection cavity; and (3) systemic targeted alpha therapy, which seeks to overcome blood-brain barrier limitations through intravenously administered radiopharmaceuticals. These approaches differ in their mechanisms of delivery, biological rationale, stage of clinical development, and clinical applicability (**Figure 3**).

## 4 Interstitial alpha therapy: alpha DaRT

**Figure 4:**
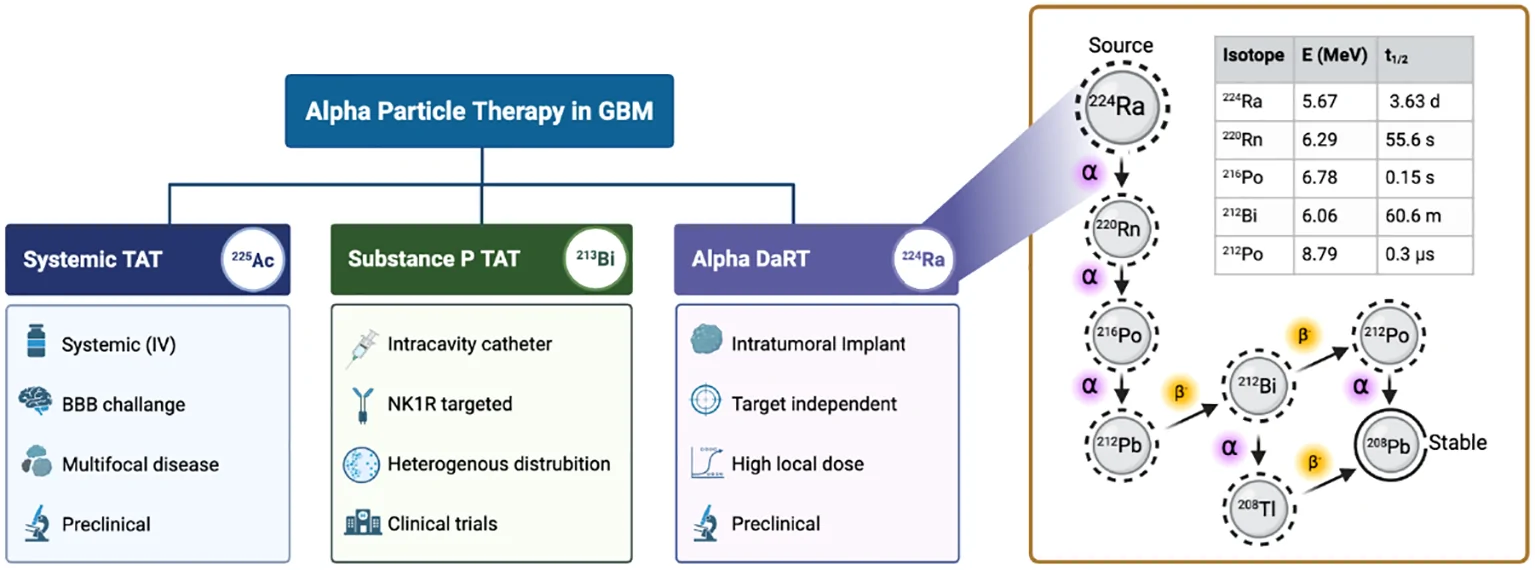
Current alpha particle therapeutic strategies under investigation for glioblastoma. Created in BioRender. Atak, E. (2026) https://BioRender.com/1tgzyat.

Alpha DaRT utilizes ^224^Ra containing sources (half-life 3.66 days) implanted directly into tumor tissue. Unlike conventional brachytherapy sources that confine radiation delivery to a limited radius surrounding the implant, Alpha DaRT releases short lived alpha emitting daughter isotopes, including ^220^Rn, ^212^Pb, and ^212^Bi, which diffuse through the surrounding tissue and extend the therapeutic range beyond the source itself (25, 26) (**Figure 4**). Dosimetric modeling demonstrated that therapeutically relevant alpha particle doses can be achieved within a region measuring approximately 4 to 7 mm in diameter around each source (25). When multiple sources are arranged in a hexagonal lattice with approximately 4-mm spacing, effective tumor coverage can be achieved while maintaining steep dose falloff to adjacent normal tissue (27). This delivery strategy may be particularly advantageous for recurrent glioblastoma, where additional external beam irradiation is frequently constrained by prior radiation exposure. However, the limited range of alpha particles represents both strength and limitation. Although the highly localized dose distribution minimizes radiation exposure to adjacent normal brain tissue, infiltrative glioblastoma cells often extend beyond the radiographically visible lesion and may lie outside the effective treatment radius of implanted sources. Consequently, careful patient selection, optimization of source spacing and implantation geometry, and integration with other treatment modalities may be necessary to maximize tumor coverage while preserving the dosimetric advantages of Alpha DaRT. To facilitate accurate intracranial delivery, Alpha DaRT incorporates a dedicated stereotactic implantation platform that enables controlled radial deployment of multiple ^224^Ra-containing sources. Source length and configuration can be tailored to tumor geometry, and image-guided stereotactic navigation is used to optimize source placement and confirm implantation accuracy. This integrated workflow is designed to maximize target coverage while maintaining safe distances from adjacent organs at risk and eloquent brain structures (**Figure 3**).

Clinical experience with Alpha DaRT has been limited primarily to extracranial malignancies. In a pooled analysis of four prospective studies including 81 lesions in 71 patients with head and neck or skin cancers, complete response was achieved in 89% of treated lesions, with a two-year actuarial local recurrence free survival of 77% (28). Importantly, no grade 2 or greater late toxicities were reported. Similar findings were observed in the first multicenter human trial, in which 78.6% of squamous cell carcinoma lesions achieved complete response without grade 3 or higher treatment related toxicities (29). Recently, Alpha DaRT was evaluated in patients with advanced pancreatic cancer using an endoscopic ultrasound guided approach. Across three prospective studies involving 58 patients, treatment achieved an 87% disease control rate, a 27% objective response rate, and a 7.9 month median OS, with no treatment related mortality reported (30). Although these studies demonstrate the technical feasibility and favorable safety profile of Alpha DaRT in humans, their efficacy outcomes should not be extrapolated to glioblastoma because of the unique anatomical, biological, and therapeutic challenges associated with intracranial disease.

Although clinical experience in glioblastoma remains limited, preclinical studies provide an important proof of concept. Nishri and colleagues demonstrated significant antitumor activity of Alpha DaRT in mice bearing human U87 glioblastoma xenografts (21). The combination of Alpha DaRT and temozolomide produced substantially greater cytotoxicity than either treatment alone *in vitro* and delayed tumor growth more effectively than monotherapy *in vivo*. Notably, alpha irradiation did not increase VEGF secretion, a response commonly observed following conventional radiation exposure and implicated in tumor angiogenesis and recurrence. Furthermore, the addition of bevacizumab enhanced tumor control and increased the intratumoral diffusion of radium progeny while reducing systemic clearance, suggesting a potentially favorable interaction between antiangiogenic therapy and Alpha DaRT.

Several features make Alpha DaRT particularly attractive for glioblastoma treatment. The short path length of alpha particles minimizes radiation exposure to adjacent normal brain tissue, while the high LET produces potent cytotoxic effects that are largely independent of oxygenation status and cell cycle phase. Continuous dose delivery over several weeks may provide sustained biological pressure against residual tumor cells within the postoperative cavity and infiltrative tumor margins. In addition, accumulating evidence suggests that Alpha DaRT may interact favorably with systemic therapies, including temozolomide, antiangiogenic agents, and immunotherapy, providing opportunities for multimodality treatment strategies (21, 31, 32). Although both Alpha DaRT and targeted alpha therapy utilize high-LET alpha radiation, they represent fundamentally different therapeutic and dosimetric paradigms. Alpha DaRT is an interstitial brachytherapy platform in which implanted ^224^Ra-containing sources continuously release short-lived alpha-emitting daughter radionuclides that diffuse locally through surrounding tissue, resulting in highly localized dose deposition that is independent of molecular target expression. In contrast, ligand-based targeted alpha therapy relies on selective binding of radiolabeled ligands to tumor-associated receptors, with therapeutic efficacy influenced by target expression, radiopharmaceutical pharmacokinetics, chelator stability, radionuclide retention, and heterogeneous intratumoral uptake. Consequently, these approaches differ substantially in their mechanisms of delivery, dosimetry, biological requirements, and potential clinical applications.

## 5 Locoregional targeted alpha therapy: radiolabeled substance P

A complementary approach to alpha particle delivery exploits the overexpression of neurokinin type-1 receptors on glioblastoma cells (33, 34). Substance P, the endogenous ligand for this receptor, was radiolabeled with alpha emitting isotopes including ²¹³Bi and ²²^5^Ac and administered directly into the resection cavity or tumor bed (35–37). Clinical experience with radiolabeled Substance P is currently limited to early-phase, non-randomized studies primarily designed to evaluate the feasibility, safety, and dosimetry of locoregional targeted alpha therapy. In a cohort of 20 patients with recurrent glioblastoma treated with ²¹³Bi DOTA Substance P, treatment was generally well tolerated and associated with only mild transient adverse effects (36). Median progression free survival was 2.7 months, median OS from initial diagnosis was 23.6 months, and median OS after recurrence was 10.9 months. Subsequent dose escalation studies using ²²^5^Ac DOTA Substance P confirmed the feasibility of administering up to 30 MBq per treatment cycle, with median OS reaching 35.0 months from diagnosis and 13.2 months following recurrence (35). Particularly favorable outcomes were reported among patients with secondary glioblastoma, suggesting that specific molecular subgroups may derive greater benefit from targeted alpha therapy (23, 38). However, these findings should be considered hypothesis-generating. Although encouraging survival outcomes were reported, these findings should be interpreted cautiously because the studies were small, non-randomized, and potentially influenced by patient selection, molecular subtype, prior therapies, treatment timing, and concurrent management. Therefore, the available data should be considered preliminary and hypothesis-generating rather than definitive evidence of clinical efficacy.

## 6 Systemic targeted alpha therapy: emerging systemic approaches

While most alpha particle strategies for glioblastoma focused on local or intracavitary delivery, systemic approaches are also under active investigation. One of the principal barriers to systemic radionuclide therapy in brain tumors is the blood brain barrier, which limits tumor uptake of circulating therapeutic agents. To address this challenge, Nair and colleagues developed ²²^5^Ac labeled dendrimer radio conjugates and combined treatment with low dose temozolomide to transiently increase tumor vascular permeability (39). In an orthotopic GL261 glioma model, the combination significantly improved survival compared with radio conjugates alone and substantially outperformed temozolomide monotherapy and untreated controls.

These therapeutic platforms illustrate the evolution of alpha particle therapy from proof-of-concept studies to increasingly sophisticated approaches tailored to the biological characteristics of glioblastoma. While Alpha DaRT enables highly localized interstitial irradiation of infiltrative tumor margins, radiolabeled Substance P exploits tumor-specific receptor expression, and emerging systemic approaches seek to overcome the limitations imposed by the blood-brain barrier. Together, these strategies provide complementary approaches to induce complex DNA damage, target GCS populations, reduce the impact of hypoxia-associated radio resistance, and potentially remodel the tumor immune microenvironment, providing the biological rationale for the biomarker-focused discussion presented in the following section. The major alpha-emitting therapeutic platforms currently under investigation for glioblastoma are summarized in **Table 1**.

**Table 1:** Current alpha particle therapeutic platforms for glioblastoma: clinical development, key findings, advantages, and limitations.

Delivery platform	Therapy	Target	Evidence level	Key findings	Advantages	Limitations
Targeted α therapy	²¹³Bi-DOTA-Substance P (36–38)	NK1 receptor	Clinical GBM Phase I/II	Safe with mPFS 2.7 months and mOS 10.9 months after recurrence	Selective NK1R targeting; high LET; minimal toxicity	Short half-life; limited intratumoral diffusion; catheter placement required
	²²^5^Ac-DOTA-Substance P (35, 40)	NK1 receptor	Clinical GBM Phase I/II	Dose escalation demonstrated safety with mOS 13.2 months after recurrence	Longer half-life; multiple α emissions; prolonged tumor irradiation	Daughter redistribution; limited isotope availability; catheter placement required
	²¹¹At-ch81C6 (41)	Tenascin-C	Clinical GBM Phase I	Favorable survival with no dose-limiting toxicity	No long-lived daughters; imaging capability; localized irradiation	Short half-life; cyclotron production; variable tenascin expression
Interstitial α therapy	Alpha DaRT (21, 22)	Target-independent	GBM Preclinical	Delayed GBM growth and synergistic activity with temozolomide and bevacizumab	Bypasses BBB; highly localized high-LET irradiation; receptor independent	Requires implantation; limited to localized tumors; no human GBM data
Systemic α therapy	²²^5^Ac-dendrimer (39)	Passive tumor accumulation	GBM Preclinical	Improved survival with TMZ-mediated BBB modulation	Systemic administration; enhanced tumor uptake after TMZ	BBB remains a major challenge; preclinical only
	²²^5^Ac-E4G10 (42, 43)	VE-cadherin	GBM Preclinical	Improved survival with vascular remodeling and immune modulation	Targets tumor neovasculature; may improve drug delivery	Preclinical only; potential vascular off-target effects
	²²^5^Ac-Pep-1L (44, 45)	IL13RA2	GBM Preclinical	Increased survival with enhanced DNA damage	Targets IL13RA2 expressed in most GBMs; potent antitumor activity	Requires convection-enhanced delivery; heterogeneous target expression
	²¹¹At-FAPI/RGD (46–48)	FAPα/Integrins	GBM Preclinical	Significant tumor reduction and prolonged survival	Rapid tumor penetration; stromal targeting	Short half-life; preclinical only
	²¹¹At-9E7.4 (49)	Syndecan-1	GBM Preclinical	Durable survival and induction of immune memory	Strong immunogenic effects; long-term tumor control	Preclinical only; immunogenic antibody; variable target expression

α, alpha; Ac, actinium; At, astatine; BBB, blood–brain barrier; Bi, bismuth; DOTA, tetraazacyclododecane tetraacetic acid; FAPI, fibroblast activation protein inhibitor; FAPα, fibroblast activation protein alpha; GBM, glioblastoma; IL13RA2, interleukin-13 receptor alpha 2; LET, linear energy transfer; mOS, median overall survival; mPFS, median progression-free survival; NK1R, neurokinin-1 receptor; TMZ, temozolomide; VE-cadherin, vascular endothelial cadherin.

## 7 Biological mechanisms of response and emerging biomarkers of alpha particle therapy

### 7.1 Direct DNA damage and DNA damage response

The therapeutic efficacy of alpha particle therapy is primarily driven by the induction of complex and densely clustered DNA damage. Unlike conventional photon irradiation, which generates sparsely distributed DNA lesions through indirect free radical formation, alpha particles deposit large amounts of energy over extremely short distances, resulting in multiple double strand breaks within localized regions of chromatin (15). These clustered lesions are difficult to repair and frequently lead to chromosomal instability, mitotic catastrophe, apoptosis, and permanent loss of clonogenic potential (50).

DNA damage response pathways represent some of the most promising biomarker candidates for alpha particle therapy because they are directly linked to both treatment mechanism and tumor progression. Aberrant activation of DNA repair pathways contributes to radio resistance, genomic instability, and disease recurrence in glioblastoma. These biomarkers are best considered pharmacodynamic biomarkers because they reflect treatment-induced DNA damage and activation of DNA repair pathways rather than established pretreatment predictors of therapeutic benefit. Consequently, biomarkers reflecting treatment-induced DNA damage may provide valuable pharmacodynamic indicators of therapeutic activity while simultaneously identifying resistance mechanisms that influence clinical outcomes. Among the most extensively studied markers is γ-H2AX, which accumulates at sites of DNA double strand breaks (51). Following alpha irradiation, γ-H2AX foci persist considerably longer than after low LET radiation, with reported half-lives ranging from 12.5 to 16.9 hours, reflecting the relative irreparability of alpha induced DNA damage (52). Additional candidates include 53BP1, ATM activation signatures, and RAD51 expression (15, 53). RAD51 is of particular interest in glioma because elevated expression was associated with radio resistance and enhanced homologous recombination repair capacity (54). Persistent activation of these pathways following alpha radiation may serve as a biomarker of treatment-induced DNA damage and may provide insight into intrinsic resistance mechanisms. However, their value as predictive biomarkers for treatment selection remains unproven and requires prospective evaluation in studies of specific alpha-emitter platforms.

### 7.2 Targeting GSCs

GSCs are increasingly recognized as a major driver of treatment resistance and disease recurrence. These cells possess self-renewal capacity, enhanced DNA repair mechanisms, and the ability to repopulate tumors following surgery, chemotherapy, and radiotherapy (55). Their preferential localization within hypoxic niches further contributes to resistance against conventional treatment approaches. Several stemness-associated biomarkers, including CD133, CD44, SOX2, Nestin, and RAD51 were linked to aggressive disease behavior, poor prognosis, and resistance to therapy (56, 57). Because alpha radiation generates clustered DNA damage that is less dependent on conventional repair pathways, alpha-emitting therapies may be capable of overcoming some mechanisms of GSC-mediated radio resistance (58). Although direct evidence in glioblastoma remains limited, these biomarkers remain biologically plausible candidate predictive biomarkers that require prospective clinical validation before they can be used for treatment selection.

### 7.3 Hypoxia independent cytotoxicity

Tumor hypoxia represents one of the most important determinants of treatment resistance in glioblastoma. Hypoxic regions promote genomic instability, angiogenesis, tumor invasion, and maintenance of GSC phenotypes while simultaneously reducing the effectiveness of conventional radiotherapy through diminished oxygen-depending free radical formation. Consequently, hypoxia contributes substantially to tumor progression and poor clinical outcomes. A major advantage of alpha particle therapy is its relative independence from tumor oxygenation status. The densely ionizing radiation tracks generated by alpha particles produce direct DNA damage that is less reliant on oxygen mediated chemical reactions (35). Consequently, alpha irradiation may retain efficacy within hypoxic tumor regions. Several hypoxia-associated biomarkers, including hypoxia-inducible factor-1α (HIF-1α), carbonic anhydrase IX (CAIX), vascular endothelial growth factor (VEGF), hypoxia-related gene expression signatures, and functional imaging approaches, demonstrated prognostic significance in glioblastoma. These biomarkers are established prognostic markers in glioblastoma and may help identify tumors that are particularly dependent on hypoxia-driven progression. Whether they predict response to alpha particle therapy remains unknown and requires prospective validation.

### 7.4 Immunogenic cell death and tumor microenvironment remodeling

Beyond direct tumor cell killing, alpha particle therapy appears capable of reshaping the tumor microenvironment through induction of immunogenic cell death (42). This process is characterized by calreticulin exposure on the cell surface, extracellular ATP release, and secretion of HMGB1, all of which function as damage associated molecular patterns that promote dendritic cell recruitment and activation (59). Preclinical studies demonstrated that alpha emitting radionuclides can increase MHC class I expression and enhance antigen presentation (60). These changes may facilitate recognition of tumor associated antigens and promote the development of adaptive antitumor immunity (61). Such effects are particularly important in glioblastoma, which is typically characterized by profound local and systemic immunosuppression. Alpha radiation also activates multiple immune signaling pathways. Radiation induced DNA damage results in accumulation of cytosolic DNA fragments that stimulate the cyclic GMP AMP synthase and stimulator of interferon genes pathway, leading to production of type I interferons and enhanced dendritic cell maturation (31, 62, 63). In addition, it induces expression of chemokines including CXCL9, CXCL10, CXCL11, and CXCL16, which recruit cytotoxic T cells and natural killer cells into the tumor microenvironment (31).

Emerging evidence suggests alpha particle therapies may produce more robust immune activation than beta emitting radiopharmaceuticals. Preclinical studies demonstrated increased CD8 to regulatory T cell ratios, enhanced activation of effector and memory T cells, depletion of suppressive myeloid populations, and upregulation of proinflammatory cytokines following alpha particle treatment (63, 64). Clinical observations from Alpha DaRT studies similarly suggested preservation of systemic immune function and reduction of inflammatory cytokines such as IL-6, findings that differ from the lymphopenia frequently observed following external beam radiotherapy (31). Several immune-related biomarkers may prove valuable for monitoring response to alpha particle therapy. These biomarkers should be considered candidate response-monitoring biomarkers that may provide insight into treatment-induced immune remodeling rather than validated predictors of treatment response. Candidate markers include CD8^+^ T-cell infiltration, regulatory T-cell abundance, PD-L1 expression, interferon signaling signatures, circulating cytokine profiles, and measures of cGAS-STING pathway activation. These biomarkers may provide mechanistic insight into treatment-induced immune remodeling and support the development of rational combination strategies with immunotherapy.

### 7.5 Molecular biomarkers of response

Several molecular features may influence sensitivity to alpha particle therapy. MGMT promoter methylation is the most well-established predictive biomarker for benefit from temozolomide in glioblastoma but has not been validated as a predictive biomarker for alpha-particle therapy. Current evidence suggests that the biological effects of alpha radiation are largely independent of MGMT status (52). Preclinical studies indicate that the combination of temozolomide and alpha radiation may produce enhanced cytotoxicity in MGMT methylated tumors, highlighting the potential importance of biomarker driven combination approaches; however, these findings remain exploratory and require clinical validation before informing biomarker-driven treatment strategies (21, 52).

Additional candidate biomarkers include IDH1 and IDH2 mutation status, TP53 alterations, PTEN loss, EGFR amplification, CDKN2A/B deletion, and abnormalities involving homologous recombination repair pathways. Most of these molecular alterations are established prognostic biomarkers in glioblastoma or predictive biomarkers for other treatment modalities, but their predictive significance for alpha-particle therapy has not yet been established. Many of these molecular alterations influence tumor evolution, genomic instability, and therapeutic resistance and may therefore affect sensitivity to alpha irradiation. Most currently available biomarkers were developed in the context of chemotherapy or conventional external beam radiotherapy, and it remains uncertain whether these relationships can be extrapolated to high-LET alpha radiation.

## 8 Discussion

Alpha particle therapy possesses attractive radiobiological characteristics that provide a compelling mechanistic rationale for investigation in several biological pathways contributing to glioblastoma therapeutic resistance. Although clinical experience remains limited, emerging preclinical and early clinical data indicate that alpha particle therapies may complement existing treatment approaches while providing opportunities for biomarker-guided patient selection and treatment monitoring (21, 22, 38). Many biological pathways that drive glioblastoma progression, including enhanced DNA damage repair, GSC persistence, hypoxia-driven adaptation, and immune escape, may also influence response to alpha particle therapy. This convergence of tumor biology and therapeutic vulnerability provides a strong rationale for the development of biomarker-guided treatment strategies (55, 65, 66). However, whether biomarkers validated in the context of chemotherapy or conventional radiotherapy retain predictive value for high-LET alpha radiation remains largely unknown and warrant investigation.

Beyond its biological advantages, Alpha DaRT may also address an important clinical challenge in recurrent glioblastoma. Conventional salvage reirradiation is frequently constrained by cumulative radiation dose to surrounding normal brain tissue, limiting treatment options for many patients (67). Owing to the short path length and high linear energy transfer of alpha particles, Alpha DaRT has the potential to deliver highly localized radiation while minimizing additional exposure to previously irradiated normal tissue, providing a strong rationale for its investigation in recurrent disease. Although conventional local dose-escalation strategies, including intraoperative radiotherapy in the INTRAGO-II trial, did not demonstrate improved clinical outcomes, the distinct radiobiological properties of high-LET alpha particles provide a rationale for investigating focal alpha-particle boost strategies in recurrent glioblastoma (68). Among currently available biomarker candidates, DNA damage response pathways appear particularly promising. Alterations involving homologous recombination repair, ATM signaling, and RAD51-mediated repair was associated with radio resistance and poor clinical outcomes in glioma (69–71). Because alpha particles generate highly complex DNA lesions that challenge cellular repair mechanisms, tumors characterized by deficiencies in these pathways may demonstrate enhanced sensitivity to treatment (15). Conversely, persistent activation of DNA repair signaling following therapy may identify resistant subpopulations capable of driving disease recurrence. Integration of genomic and functional DNA repair assessments into future clinical studies could therefore facilitate both patient stratification and mechanistic understanding of treatment response. GCS-associated biomarkers represent another area of considerable interest. Stemness-related markers such as CD133, CD44, SOX2, Nestin, and RAD51 were associated with treatment resistance and recurrence (66, 72, 73). The possibility that alpha particle therapy may be effective in the setting of GSC-mediated radio resistance is particularly attractive given the central role of GSCs in disease progression. Future studies should determine whether these biomarkers identify tumors that are especially susceptible to alpha irradiation and whether treatment can effectively deplete GSC populations responsible for tumor repopulation.

The relationship between alpha particle therapy and the tumor microenvironment has important translational implications. Predominantly preclinical evidence suggests that alpha radiation induces immunogenic cell death and activates innate and adaptive immune pathways capable of modifying the local immune landscape (32, 74). Such effects may be particularly relevant in glioblastoma, where profound immunosuppression contributes to therapeutic failure. However, the magnitude of these immune effects is likely to vary according to the alpha-emitter platform, dose distribution, tumor model, treatment sequence, and combination regimen. Biomarkers reflecting immune activation, including interferon signaling signatures, cGAS-STING pathway activity, cytokine profiles, and measures of immune cell infiltration, may provide valuable insight into treatment-induced microenvironmental remodeling (75). These observations further support investigation of rational combination strategies incorporating immune checkpoint blockade or cellular therapies.

Beyond predictive biomarkers, the development of reliable response-monitoring tools represents a major unmet need. Radiographic assessment following treatment remains challenging because treatment-related changes can closely resemble tumor progression (76). Liquid biopsy technologies, including cerebrospinal fluid-derived circulating tumor DNA, extracellular vesicles, circulating microRNAs, and cell-free DNA fragmentation profiles, offer promising opportunities for noninvasive response monitoring, early detection of residual or recurrent disease, and longitudinal surveillance following treatment (77). Similarly, advanced imaging approaches such as amino acid PET, diffusion-weighted MRI, perfusion imaging, and radiomic analyses may improve early response assessment, facilitate differentiation of pseudoprogression from true tumor progression, and support surveillance for disease recurrence following alpha-particle therapy. For radiomic models intended to support clinical decision-making, performance should be evaluated beyond discrimination metrics alone and include calibration, clinical utility (e.g., decision-curve analysis), and model interpretability to facilitate reliable translation into clinical practice (78, 79). The integration of molecular and imaging biomarkers may ultimately enable more precise assessment of treatment efficacy while facilitating earlier detection of recurrence and supporting biomarker-guided precision oncology approaches (80).

The growing body of evidence supporting Alpha DaRT in both preclinical and clinical settings further strengthens its translational potential for glioblastoma. Preclinical studies demonstrated that localized high-LET alpha radiation can achieve potent tumor control while potentially overcoming hypoxia-associated radio resistance and targeting infiltrative tumor cells beyond the immediate implantation site (21, 22, 81). In parallel, early clinical studies in recurrent cutaneous squamous cell carcinoma and other solid tumors demonstrated that Alpha DaRT implantation is technically feasible, well tolerated, and capable of achieving encouraging local control (28, 29, 82). Although glioblastoma presents unique anatomical and biological challenges, these findings provide important proof-of-concept for interstitial alpha-particle therapy and support continued investigation of Alpha DaRT as a treatment strategy for recurrent disease (17). Beyond demonstrating biological efficacy, successful clinical implementation of alpha-particle therapy will also depend on accurate dosimetry and optimized treatment delivery. The highly localized dose deposition of alpha emitters makes therapeutic efficacy particularly sensitive to source placement geometry, intersource spacing, and heterogeneous intratumoral dose distribution. For interstitial approaches such as Alpha DaRT, additional challenges include diffusion and clearance of radioactive daughter radionuclides, and maintaining adequate target coverage while minimizing radiation exposure to adjacent normal brain tissue.

Despite these opportunities, several barriers must be overcome before alpha particle therapy can be incorporated into routine glioblastoma management. Clinical evidence remains limited, particularly for Alpha DaRT, where intracranial human experience has only recently begun. The first clinical trial evaluating Alpha DaRT in GBM (NCT06910306) is currently recruiting patients with recurrent glioblastoma not amenable to surgical resection. This ongoing early feasibility and safety study enrolls patients with a single recurrent lesion measuring ≤3 cm and evaluates technical feasibility of source implantation, radiation dosimetry, treatment-related toxicity, and preliminary clinical outcomes, including radiographic response, pseudoprogression, local tumor control, and OS. In addition, the protocol incorporates comprehensive neurological assessments, serial MRI evaluation, radiation dosimetry, and radiation safety monitoring, providing an important foundation for future efficacy trials and biomarker-driven clinical development. Uncertainties regarding radionuclide distribution, postoperative cavity dynamics, source placement, and patient-specific dosimetry remain important challenges. In addition, the intracranial environment presents unique challenges that are not encountered in extracranial tumors, including prior radiation exposure, infiltrative tumor growth, treatment-related edema, proximity to eloquent brain structures, and the risk of neurological toxicity. Addressing these issues will require prospective clinical trials incorporating standardized dosimetric methodologies, MRI-based response assessment, comprehensive neurological and toxicity evaluation, molecular profiling, serial cerebrospinal fluid and plasma biomarker sampling, and prespecified translational endpoints. Such integrated trial designs will be essential to determine whether candidate biomarkers are truly predictive of response to alpha-particle therapy rather than simply prognostic or reflective of treatment exposure.

In contrast to interstitial approaches, the clinical translation of systemically administered targeted alpha therapies presents additional challenges. Blood-brain barrier penetration may limit radiopharmaceutical delivery to infiltrative tumor cells, while heterogeneous target expression and variable intratumoral uptake can result in nonuniform radiation dose distribution (83, 84). Practical considerations, including isotope availability, chelator stability, and daughter radionuclide recoil or redistribution, may further influence therapeutic efficacy and dosimetry. In addition, off-target radiation exposure to dose-limiting organs, particularly the bone marrow and kidneys, remains an important consideration for systemically administered alpha emitters (85). Continued optimization of radiopharmaceutical design, patient selection, and individualized dosimetric approaches will be critical for the safe and effective clinical translation of targeted alpha therapies for glioblastoma.

Overall, alpha particle therapy remains an investigational therapeutic strategy for glioblastoma. Current evidence supports a strong preclinical rationale and early clinical feasibility, but routine clinical implementation and biomarker-directed patient selection remain premature. Nevertheless, alpha particle therapy represents more than a novel radiation delivery platform; it provides a framework for exploring biomarker-driven therapeutic strategies in glioblastoma. Future progress will depend not only on demonstrating clinical efficacy but also on identifying the biological determinants of response and resistance. Prospective integration of molecular profiling, liquid biopsy technologies, immune monitoring, and advanced imaging into clinical trial design will be essential for defining the role of alpha particle therapies within precision oncology approaches for glioblastoma.
